# Emergency centre patients in the Democratic Republic of Congo: Some epidemiological and clinical aspects at Beni General Referral Hospital

**DOI:** 10.1016/j.afjem.2023.01.001

**Published:** 2023-01-18

**Authors:** Agnes K Katsioto, Pascaline K Muhesi, Job P Isombi, Prosper K Kambere, Franck K Sikakulya

**Affiliations:** a Emergency Medicine Department, Alexandria University Faculty of Medicine, Egypt; b Faculty of Medecine, Catholic University of Graben, DRC; c Faculty of Medecine, University of Goma, DRC; d Pediatrics Department, Kampala International University Western Campus, Uganda; e Faculty of Eenvironmental and agronomic sciences, Université Officielle du Semuliki, DRC; f General Surgery Department, Faculty of Medecine, Kampala International University Western Campus, Uganda

**Keywords:** Attendance, Democratic Republic of Congo, Emergency centre, Epidemiology

## Abstract

**Introduction:**

Little information is available regarding the characteristics of patients attending the emergency centre (EC) in the Democratic Republic of Congo. This study aims to provide some epidemiological and clinical aspects of patients admitted to the emergency centre at Beni General Referral Hospital.

**Methodology:**

For a year, from January to December 2021, a cross-sectional study was conducted. Data regarding patients’ characteristics, admission modality, stay duration, reason for admission, and discharge modality was anonymously collected from patients’ registers. A descriptive analysis was done with Epi-Info 7.

**Result:**

A total of 1404 patients were admitted to the EC, with a male-to-female ratio of 1.2 to 1. The age group below 18 years accounted for 35.4%. Most of the patients (75.7%) originated from urban areas. In 83% of cases, there was no recommendation from another medical facility for EC admission. The most common reasons for admission are non-traumatic gathering on top of neuropsychiatric and non-specific symptoms. Road traffic accidents are the most frequent causes of trauma symptoms. Few patients (14.7%) spent less than 12 hours in the EC. Globally, 7.3% of patients admitted to the EC were discharged after being managed, and 89% were transferred to different wards. The intra-emergency centre mortality rate was 11.8% among admitted patients in the ER at Beni General Referral Hospital.

**Conclusion:**

This epidemiology database underlines the need for developing globalizing and multi-sectoral interventions (diagnosis, therapeutic strategy, organization, health program, or health policies) in the perspective of bringing change and/or taking action in the Democratic Republic of Congo's emergency medical system.

## African relevance

• There is a lack of epidemiological data in sub-Saharan countries. A description of emergency centre attendance in the Democratic Republic of the Congo, with its specific tropical conflict settings, is needed. It helps to get an idea about the impact of complex emergencies on health.
• Public health programs, decision-makers, and researchers rely on existing or known facts to plan their activities relating to the health of the population.
• We emphasize the critical importance of implementing good practices in emergency medicine in the Democratic Republic of Congo and other African countries where they do not yet exist.

## Introduction

A medical emergency is described as the perception of any situation that is rapidly deteriorating or likely to do so, either without or with medical care. [1]. However, the World Health Organization (WHO) does not really give definitions of an emergency but cites the right to health care and emergency medical care and defines a health emergency as a phenomenon that occurs suddenly and unexpectedly, which surprises and worries, positively or wrongly; the person concerned and, or those around him but also as an unforeseen situation, of sudden onset and requiring a rapid response [2].

Emergency centres (EC) around the world are a vital link between pre-hospital care and hospitalization [3]. Over the past few decades, hospital EC have steadily increased in activity [4]. This constant increase in the use of emergency services is a phenomenon common to all countries; mainly developed countries [5,6].

Patient care in EC is one of the best showcases of a health system. According to a study, the reasons for choosing the EC were mainly motivated by the quickness of action (54.0%), easy accessibility (47.7%), followed by acute features of the illness (26.4%) [7]. In developing countries, emergency care management faces human and material shortages [8].

Despite the disproportionately high incidence of acute sickness and injury in Sub-Saharan Africa, few clinical facilities are set up to employ an integrated strategy for triage, stabilization, and resuscitation. [9] Emergency medicine is still a rudimentary field in the Democratic Republic of the Congo (DRC). Most hospitals do not have an emergency department, and if one exists, it is just reduced to an emergency reception room [10]. This tropical country is permanently facing many complex emergencies (environmental disasters, epidemic outbreaks, war, hunger, population displacement, etc.), leading to several health emergencies [11]. They require urgent admission and management within nearby health facilities.

Little information is available regarding the characteristics of patients attending the emergency centre in the DRC. When these details could allow a rational organization and a compelling adaptation of the care supply according to the nature of the demand. Such data has tremendous potential to support more efficient health service planning and delivery, help clinicians monitor patient safety, improve treatments and prevention, and advance the understanding of disease. This study aims to describe some clinical and epidemiological characteristics of patients who attended the emergency centre of Beni General Referral Hospital (GRH).

## Methods

This cross-sectional study was performed for a period of one year; from 1st January to 31st December 2021.

It was conducted at Beni GRH, a tertiary healthcare facility that serves as a referral centre for hundreds of thousands of people living in Beni town and neighbourhood, in the East of DRC, in the central Africa. This region is facing army and civilian conflicts in the country since more than three decades. This health structure offers care to patients with medical and surgical diseases according to the hospital abilities. The emergency centre has six beds and it is opened every day 24/7. The staff shared with the intensive care unit includes two medical officers, ten nurses and two hygienists. No triaging system or ambulance service is available. Point-of-care tests and technologies are rarely accessible. New generations of emergency and lifesaving treatments are hardly available; and the practice of medical technics faces significant limitations due to lack of materials and skilled personnel. Furthermore, the accessibility to healthcare (treatment, laboratory exams and imaging) requires a payment.

Our study population includes all patients who visited the emergency centre during the study period. Admitted in wards after outpatient consultations, patients admitted in neonatology and obstetrics unities were omitted, because they are not seen at the emergency centre. Lifeless patients at admission; patients who refused care, patients seeking a simple medical prescription or para-clinic results interpretation or vital signs assessment were also excluded. Our sampling was exhaustive and non-random including patients whose data were well and fully completed.

Reasons for admission to the EC were subdivided into traumatic and non-traumatic. Traumatic reasons were classified according to the trauma mechanisms. Referring to previous similar studies [12], [13], [14], non-traumatic reasons were globalized and classified according to anatomic systems.

The time spent in the EC, from physical entry to exit (discharge), was used to determine the length of stay. There is no regulation on the amount of time a patient can spend in the EC.

Disposition options from the EC included discharge to home, admission to a hospital ward, transfer to an intensive care unit (ICU), transfer to other health facilities, and death within the EC.

Data entry was performed using Microsoft Excel software, then exported to Epi Info 7.2.5 for analysis. The frequencies and proportions of parameters were recorded, calculated, and reported as rates.

The study was carried out after receiving approval from the review board of the *Université Officielle du Semuliki* and the Beni General Referral Hospital. The patient's names were omitted during data collection. The data was stored on a private computer with a password known only to the researcher.

## Results

### Socio-demographic characteristics

During our study period, a total of 1404 patients who met our inclusion criteria were admitted to the emergency centre. A male-to-female ratio of 1.2/1 was discovered. The participants' ages ranged from 1 to 98 years, with a median age of 29.3 years. Children under the age of ten and elderly patients over the age of 55 accounted for 20.9% and 18.1% of all EC admissions, respectively; globally, the EC frequency rate decreases gradually as one gets older and suddenly rises during the fifties. Most of the patients (75.7%) originated from Beni City (Table 1).

**Table 1:** Socio-demographic characteristics of patients admitted to emergency centre.

Variables	Frequency(n:1404)	Percentage (%)
Age (years)		
[1–9]	298	21.2
[10–18]	204	14.5
[19–27]	178	12.7
[28-36]	151	10.8
[36-45]	163	11.6
[45-54]	158	11.1
[55-63]	117	8.3
[64-72]	86	6.0
[73 and above]	53	3.8
Gender		
Male	770	54,8
Female	634	45,1
Address		
Beni city	1063	75,7
Outside Beni	341	24,3

### Modality of admission

**Fig. 1:**
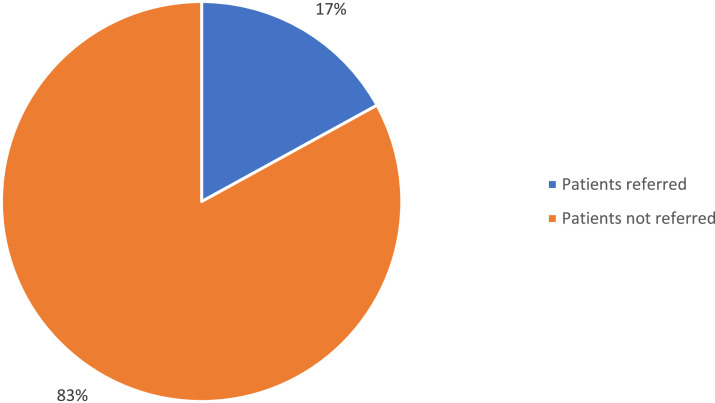
Mode of admission into the EC

Most patients (83%) were admitted without a referral form from primary health centres or other facilities. (Fig. 1)

### Reasons for being admitted to the EC

The most common reasons leading to EC admission (82.1%) are non-traumatic. Among them, general and non-specific symptoms (general feeling of illness, fatigue, asthenia, malaise, weight loss or fever, loss of appetite…) and neurological or psychiatric symptoms (disturbed level of consciousness, headache, convulsions, paralysis...) account for the other half of non-traumatic symptoms leading to EC admission. Traumatic lesions were dominated by injuries due to road traffic accidents (52.1%), followed by stab wounds (12.4%) and gunshot/explosive device injuries (11.9%). (Table 2).

**Table 2:** Reasons for admission to the EC

Disorders	Frequency n=2829	Percentage (%)
**Traumatic reasons**	**(n1=507)**	**17.9**
Burns	47	9.3
Gunshot/explosives devices injuries	57	11.2
Physical aggressions	23	4.5
Falls	38	7.5
Others	12	2.4
Road traffic accidents	267	52.7
Stab wounds	63	12.4
**Non traumatic symptoms**	**(n2=2322)**	**82.1**
Neuro-psychiatric troubles	591	25.5
General and non-specific symptoms	597	25.7
Digestives disorders	383	16.5
Respiratory disorders	309	13.3
Cardiovascular disorders	304	13.1
Genito-urinary symptoms	64	2.8
ENT/ophthalmology manifestations	48	2.1
Skin/soft tissue manifestations	26	1.1

### Duration of hospitalization in the EC

Table 3 shows that only 14.5% of patients spent less than 12 hours in the EC from their arrival to their disposition; the other 38.7% stayed for 12 to 24 hours. Some patients (16.7%) spent nights (48 hours to five days) in the EC. (Table 3)

**Table 3:** Time spent in the emergency centre

Duration (hours)	Frequency	Percentage (%)
Less than 12	204	14.5
12-24	544	38.8
24-48	419	29.8
More than 48	237	16.9
**Total**	1404	100.0

### Discharge modalities from the EC

According to our findings, after examination and management, 7.3 percent of patients admitted to the EC returned home, 78.9 percent were sent to different wards (ICU, paediatrics, internal medicine, and surgery), and 2 percent were transferred to other health facilities. The mortality rate within the EC is 11.8%. (Table 4)

**Table 4:** Disposition modalities from EC

Disposition modalities from ER	Frequency	Percentage %)
To home	103	7.3
Intensive Care Unit(ICU)	212	15.1
Paediatric department	332	23.6
Internal medicine department	294	20.9
Surgery department	268	19.1
Transfer to other facilities	28	2.0
Death within EC	167	11.9
**Total**	**1404**	**1**

## Discussion

Throughout this study, we provide some epidemiological and clinical aspects of the population attending the EC of Beni General Referral Hospital. A large number of studies have shown variability in EC admissions all over the world when analysing different parameters [3], [4], [5], [6], [7], [8]. The role of socio-demographic characteristics is useful in better understanding why patients resort to emergency care. As in several previous studies, our work involves more male than female patients [6,12,14,15]. This may be justified by the fact that men have a higher risk of suffering from conditions that lead to emergency consultation, such as road traffic accidents and other injuries, toxicological situations, stroke, cardiovascular diseases, and alcohol, according to the Moore study [16].

According to our research, globally, the frequency of attending the EC increases with age, with the highest found in children under nine years old. Our results tend toward the French reality, according to which a quarter of emergencies are paediatric. Infants under one year old use the emergency centre three times more often than the general population [17]. This finding contradicts findings from Christchurch, Australia, and the United States, where older people over the age of 65 were found to be more frequent in the emergency department [18,19]. This difference in the demographic characteristics could be explained by the difference in the areas of study. In African areas, the population is young, and the morbidity and mortality rates in children are very high, especially in the tropics [20]. Almost all patients were coming from inside Beni Town, which means an urban area, as in an American study [12].

Our study shows that 23 percent of patients were referred from other health structures to Beni GRH, similarly to the Nsiamunu study findings in Kinshasa showing that 20 percent of patients are admitted to the EC with a transfer letter [21]. Previous studies showed that referral of patients by a primary care provider to a paediatric emergency department was significantly associated with greater severity of illness and resource utilization [22]. In the DRC, the absence of an emergency medical transport system is a common barrier justified by several factors, including the lack of appropriate vehicles, the inadequacy of roads, and the inability to pay for transport services [7].

In our study, neuro-psychiatric disorders (disturbed level of consciousness, convulsions, paralysis, headaches, etc.) with non-specific symptoms such as a general feeling of illness, fatigue, asthenia, malaise, weight loss or fever, and loss of appetite represent the most frequently encountered non-traumatic reason during EC admissions at Beni GRH. In a Nigerian study, neurologic manifestations were identified as the most common reason for EC consultation [23]. At the Monkole health centre in Kinshasa, malaria was the main reason for admission in 28.5% of emergency settings [24]. The predominance of these symptoms could find an explanation in the situation of complex crises experienced in the eastern part of the DRC for three decades, leading the population to live in permanent stress, which is a risk factor for several diseases such as cardiovascular diseases, stroke, and psychological disorders. WHO estimates that one in five people in war zones have mental health conditions [25]**.** On the other hand, the collection of typical troubles might reflect the high frequency of malaria in the region. It is the first important cause of morbidity and mortality in the DRC, as in other sub-Saharan countries, claiming more than 44% of all outpatient visits and 22% of deaths among children under five years [26]. Beni is not protected from this worldwide morbidity and mortality due to road traffic accidents [27,28], where they constitute 52.2 percent of traumatic reasons for EC admissions.

Even though the mortality rate for patients increased with a long stay in the emergency department [29,30], more than 80% of patients spent at least 12 hours in the EC; some stayed for five days. When the standard length of stay in the EC is 6 hours [31], Patient movement from the EC should be done without any delay after initial stabilization; sometimes, in our conditions, it is delayed because the patient cannot afford the cost of the required urgent treatment or test. In our country, where emergency medicine is still in its infancy [10], there is a lack of validated criteria and algorithms about patient flow, yet this could help to avoid an unnecessary long stay in the EC and delay time for proper management. Due to shortages in ward bed capacities, some patients admitted to the EC are sometimes denied access to inpatient units.

The mortality rate (11.8%) during our study period was higher than in other studies (2.4% in Bulgaria [32] and 2.8% in Ethiopia [33]. In the paediatric emergencies department in Mali, the fatality rate reported was 12.39% [34]. This might be associated with several factors, including poorly equipped health facilities, poorly trained staff, and the fact that patients consult late during the critical stage of their diseases, which is globally worsened by precarious health systems and limited resources.

Combining patients discharged at home (7.3%), deceased (11.8%), and transferred to other facilities (2%), we can deduce a hospitalization rate of around 78.9%. This rate is relatively high compared to what is reported in Singapore, where the percentage of hospitalized patients going through the EC is around 30% for all services combined [35]. The absence of a validated and accredited tool for patient triage in the EC of Beni GRH can explain why some patients were admitted inappropriately. Deciding about the hospitalization or discharge of patients in the EC remains a challenging and complex task for physicians, depending on clinical and non-clinical factors.

It is an original article on the epidemiological and clinical features of cases seen in the emergency centre of a low-income health facility. It was a one-year retrospective study in which all available qualified data were included. This increases the quality of representativeness. However, there are a few issues that need to be resolved. First, since the information was not available electronically, manual data extraction was difficult due to partial completeness and some missed variables. Second, only one hospital's data was used in the study. In other words, a multicentre trial would have offered more information. Furthermore, the extracted data is limited to a one-year period. Third, patients' symptoms were sometimes ambiguous and difficult to classify. Furthermore, descriptive, prospective, and analytic studies on a large number of populations and health structures are still needed to reach accurate conclusions on the epidemiological, clinical, and paraclinical concerns of emergency centre patients’ attendances.

## Conclusion

Emergency centre visits at Beni General Referral Hospital are multifaceted, affecting patients of all ages and genders with diseases of different severity levels, and they significantly give a picture of the general burden of diseases in this hospital. Emergency services' challenges must be viewed as a magnifying mirror reflecting the dysfunctions in our entire health system, both prior to, during, and after emergency care, and perhaps even more widely, our social support system. This epidemiology database underlines the need for developing globalizing and multi-sectoral interventions (diagnosis, therapeutic strategy, organization, health program, or health policies) in the perspective of bringing change and/or taking action in the Democratic Republic of Congo's emergency medical system.

## Authors’ contribution

Authors contributed as follow to the conception or design of the work; the acquisition, analysis, or interpretation of data for the work; and drafting the work or revising it critically for important intellectual content: AK 50 %, PM and FS 20% each, JI and PK 5% each. All authors approved the version to be published and agreed to be accountable for all aspects of the work.

## Dissemination of results

Results from this study were submitted and presented in the Faculty of Medicine of Université Officelle du Semuliki during a public defense. Also, they were shared with Beni General Referral Hospital for some recommendations.

## Declaration of Competing Interest

The authors declared no conflicts of interest.
